# Pain experience of people with inflammatory bowel disease: a qualitative study

**DOI:** 10.1136/bmjgast-2025-001866

**Published:** 2025-09-05

**Authors:** Charlotte Beames, Afra Azadi, Amanda C de C Williams, ADVANTAGE Consortium, Geoffrey Woods

**Affiliations:** 1 Research Department of Clinical, Educational and Health Psychology, University College London, London, UK

**Keywords:** INFLAMMATORY BOWEL DISEASE, ABDOMINAL PAIN, PSYCHOLOGY

## Abstract

**Introduction:**

People with inflammatory bowel disease (IBD) commonly experience pain, whether during active disease or remission, which interferes with daily life and major goals and causes distress. Current psychological methods of pain management draw from musculoskeletal pain interventions, but it has not been established that the musculoskeletal model is a good fit. We aimed to outline a psychological model of IBD pain.

**Methods:**

We used qualitative methods: a very open interview (Grid Elaboration Method), conducted online and transcripts analysed for themes and subthemes. 15 men and 15 women with IBD pain, recruited from a national charity, took part in 4 months to February 2024. Participants scored their average pain 5/10 and interference by pain with activity 6/10, where 10 is maximum pain or interference.

**Results:**

We extracted five inter-related themes: on the emotional impact of pain and symptoms; the challenge of pain; restrictions due to pain and other IBD symptoms; shortcomings in healthcare, particularly for pain; and poor public understanding of IBD. Although the first theme, universally endorsed, covered anxiety about the meaning of pain, we did not find the fears about physical integrity that characterise much musculoskeletal pain, nor the avoidance of physical activities based on those fears.

**Conclusion:**

We propose that further exploration is warranted of the experience of IBD-related pain and how people adjust to it. This will inform the design of better psychologically-informed interventions to help people with IBD manage their pain, independently and in partnership with healthcare.

**WHAT IS ALREADY KNOWN ON THIS TOPIC**

- Pain in people with inflammatory bowel disease (IBD) can be distressing and disabling, but psychological interventions to address this are few and of limited benefits compared with those for musculoskeletal pain.

**WHAT THIS STUDY ADDS**

- This study identifies key themes concerned with pain in IBD that do not fit well with the musculoskeletal model. The content of anxieties about pain and the implications for everyday life differs from those in musculoskeletal pain.

**HOW THIS STUDY MIGHT AFFECT RESEARCH, PRACTICE OR POLICY**

- Further elucidation of the psychology of IBD pain can provide targets for psychological intervention to improve quality of life despite pain.

## Introduction

 Inflammatory bowel disease (IBD) encompasses the inflammatory conditions ulcerative colitis (UC) and Crohn’s disease (CD). Both conditions are regularly associated with abdominal pain, diarrhoea, rectal blood loss, anaemia, weight loss and fatigue,^1^ and both are relapsing and remitting conditions in which ‘flares’ of the disease further deplete well-being.^1 2^ Aetiology of pain in IBD remains obscure, and treatment with analgesics is of limited effect and often poorly tolerated.^1^

Abdominal pain affects over 80% of people with IBD,^3^ with 30–50% also affected during disease remission,^4^ but it can also be caused by acute and treatable problems such as strictures or fistulae. Pain can also arise from extraintestinal inflammatory manifestations^2^ such as joint pain by shared mechanisms not fully understood.^5^ Chronic IBD pain was historically attributed to gut inflammation, but is often unrelated to levels of inflammatory markers; it may be at least partly due to visceral hypersensitivity such that normal gut function becomes painful.^1^ Inflammatory bowel syndrome (IBS) has some overlapping symptoms, including abdominal pain, but without inflammation; there are markedly different opinions about whether the two can co-occur or are mutually exclusive.^6^

IBD pain can limit participation in normal, particularly social, activities; limit work and career progression; affect mental health and increase healthcare use.^1 2 6 7^ There is a strong argument for pain to be assessed and managed alongside IBD treatment, but cognitive and affective factors in visceral pain are far less studied than in musculoskeletal pain, so a better understanding of the experience of IBD-related pain is needed. To improve psychological interventions for IBD-related pain,^8^ we sought to ascertain whether current psychological models of pain, developed mainly in the context of musculoskeletal problems, accurately and adequately represent IBD pain and associated problems. Psychological intervention has largely focused on improving medication adherence and modifying stress and diet. Recent extension to pain management^9^ using cognitive and behavioural methods shows improvements to quality of life and depression in IBD, but only in the short term and with no change in disease markers such as inflammation.^10^

The psychology of pain has largely been assumed to be independent of pain site/s, focusing on patients’ unhelpful beliefs or biased cognitive processing, on the basis that these underpin avoidance of activities and hence worsen disability. The activities avoided, such as work and social engagement, are beneficial for physical and mental health rather than, as feared, causing damage and/or exacerbating pain.^11^ Pain functions to reduce threat, promote defence and maintain the body’s integrity,^11^ and people with musculoskeletal pain usually articulate their fear of damage as the reason for avoiding physical strain. The focus on fear and avoidance was formulated by Vlaeyen *et al*,^11^,^13^ but, despite possible serious complications such as intestinal obstruction and the raised risk of cancer, it is not clear how dominant such fears are in IBD or to what extent they lead to avoidance of valued activities.

A qualitative study was conducted using open-ended methods, aiming to explore psychological accounts of living with IBD pain while holding in mind the question of whether the experience was well described by current psychological models of pain.

## Methods

### Design and setting

This study was part of a larger project investigating psychological models of pain in visceral disease using qualitative methods (APDP ADVANTAGE (Advanced Discovery of Visceral Analgesics via Neuroimmune Targets and the Genetics of Extreme human phenotype): protocol https://osf.io/59d6t) and had institutional Ethical Committee approval (UCL 2182/002). Adults with IBD were recruited through a large UK charity, Crohn’s & Colitis UK (https://crohnsandcolitis.org.uk/), by an advertisement on its website for adults with IBD and associated pain. The study used a recently developed qualitative method, the grid elaboration method (GEM),^14^ to elicit what people with IBD pain feel is most important and relevant, with minimal framing by researchers or requirement for a coherent narrative.^15^ This method is based on free association, aiming to elicit subjective meanings of experience with minimal imposition of concepts or of limits to personal narratives that can arise from interview questions.^14^ Participants are free to identify areas of greatest importance to them, formulated in words, phrases or drawings.

### Participants

The sample size of 30 was determined by the wider project and is in accordance with methodology^14^ and precedents.^16 17^ Equal numbers of men and women were recruited, with particular encouragement to potential participants from minoritised ethnic groups since they are under-represented in pain research.

Inclusion criteria, provided in the participant information sheet, required that participants were 18 years or over, had IBD with chronic pain (pain every day or nearly every day for at least 3 months); spoke English well enough for interview; had access to a laptop, tablet or smartphone in order to participate and had no significant cognitive impairment.

### Procedure

People responding to the advertisement were directed to further information about the study. If they wished, they then completed a Qualtrics form with inclusion criteria and contact details. The researcher contacted potential participants, answered questions, invited return of the consent form and arranged an interview online at a time convenient to the participant. Instruction on the use of Microsoft Teams for the interview was provided for those unfamiliar with it.

Interviews lasting approximately 1 hour were conducted online between November 2023 and February 2024, using Microsoft Teams. Participants were requested to have pen and paper available for the interview and asked if they preferred the camera on or off. At the start, the researcher (CB, AA, HM) introduced herself, confirmed the participant’s preferred name and requested consent to audio-recording. She emphasised that there were no correct answers, but that she wished to hear their views as experts in their conditions. At the end, participants were asked to provide details of age, sex, ethnicity, time since diagnosis and on pain intensity and interference (from the Brief Pain Inventory (BPI))^18^; they were then thanked and given the opportunity to ask questions or provide comments on the study and were told where results of the study would be posted, as dissemination would be partly through Crohn’s & Colitis UK of which they were members. No compensation was offered for participation.

### Interviews

The first step was to ask the participant to draw an empty, numbered, two by two grid; then to put in each of the four boxes one word, phrase or drawing when prompted by the interviewer’s saying. “*We are interested in learning more about your experience of IBD pain. Please share what you associate with this, using words and/or images, whatever feels most natural for you. Please include just one image/word per box.”* Participants held their completed grids to their cameras and the researcher took a screenshot. Then the participant was asked to elaborate on the idea expressed in each box, with the researcher asking only prompting questions, such as ‘*Can you tell me more about X please?*’ or ‘*You mentioned X…*.’, until the participant had nothing further to add. When all four boxes had been addressed, the researcher listed the concepts back to the participant and asked if there was anything else s/he wished to add.

Interviews were audio-recorded and transcribed soon afterwards, anonymised and potentially identifying information, such as clinic or hospital names, removed. For reflexivity, the researcher also made notes on each interview,^19^ describing general impressions and any difficulties (eg, internet connection issues), to be saved with each transcript. Interview files were permanently deleted after transcription.

### Researcher perspectives

Three researchers (CB, AA, HM) conducted the interviews (18, three and nine interviews, respectively): all had undergraduate or postgraduate degrees in psychology. A fourth researcher (AW) was involved in data analysis. Throughout the project, researchers discussed interview findings and process, considering how their beliefs and concerns influenced their reading and analytic decisions.^20^ A statement on positionality^21^ is included in the online supplemental material briefly. Two interviewers were young white British women and one a young British-Bangladeshi woman; one interviewer had CD but did not disclose this to participants unless directly asked (in one interview); AA was trained in GEM during her degree; CB and HM were trained in GEM for this project.

### Data analysis

Thematic analysis^22 23^ is used with GEM interviews.^14^ Analysis was inductive, focusing on the experience of pain for people with IBD within their individual contexts. It followed the usual steps^22^: familiarisation with data by reading transcripts; coding (using NVivo software); analysis and refinement of codes; review and revision of themes by discussion and reference back to transcripts and the start of mapping relationships between themes; finalising themes and subthemes and their connections and selecting extracts from transcripts to illustrate them.^20^

Two processes were added consistent with GEM methods.^23^ Initial codes, with meanings and quotations, were applied by CB to six transcripts, four of which were then coded by AW using the initial codes but blind to CB’s coding. After discussion of differences, several new codes were generated and others combined or refined. The coding frame was then used for the remainder of the transcripts by CB and checked by AA. Prevalence of themes and subthemes across participants was tabulated as salience in order to identify relative frequency in the sample, to better inform the provisional model of pain in IBD and to constrain the influence of researcher bias. Rigour of the analysis was maintained through regular discussion, including with the originator and other users of GEM, and by following accepted quality criteria.^22^ The analysed results were circulated to IBD researchers, clinicians and experts by experience for comments that informed the discussion. A Consolidated Criteria for Reporting Qualitative Research checklist^24^ is provided as an online supplemental file 2.

### Brief pain inventory

All participants were asked to complete the BPI^18^ after their interview, to provide background information on our sample. The BPI is a self-administered questionnaire assessing severity of pain (least, worst, current and average, scaled 0–10 where 0 is ‘no pain’ and 10 is ‘pain as bad as you can imagine’) and its interference with mood, walking ability, general activity, relationships, work, enjoyment and sleep, each scaled 0–10-point where 0 is ‘does not interfere’ and 10 is ‘completely interferes’.^25^ Pain scores can be used as variables, as can mean scores of the seven interference items.^25^

## Results

30 participants, 15 women and 15 men, were interviewed: none refused or subsequently withdrew data. Median interview length was 53 min. There were no deviations from protocol. The participant characteristics are shown in table 1.

**Table 1:** Participant characteristics (n=30)

Variable	Value
Age	18–29: 5 women, 3 men 30–39: 6 women, 5 men 40–49: 1 woman, 4 men 50–59: 2 women, 3 men > 60: 1 woman, 0 men
Ethnicity	25 identified as white British, one as Asian/Asian British, one as mixed/mixed British, one as black/black British and two as ‘other ethnic group’
IBD	Crohn’s disease: 11 men, 11 women. Ulcerative colitis: 4 men, 4 women
Years since diagnosis	Men mean 16 years, range 3–35 years; women mean 11 years, range 1–40 years.
Average pain	Mean 5/10, range 1–10
Interference by pain with activity	Mean 6/10, range 1–10.

IBD, inflammatory bowel disease.

### GEM grids

Most GEM boxes were completed with a single word or idea about symptoms and restrictions or descriptions of pain and its impact on psychological well-being; a few addressed other aspects. Only 13 out of 120 entries featured images. For examples of GEM grids, see the online supplemental material.

### Thematic analysis

**Figure 1:**
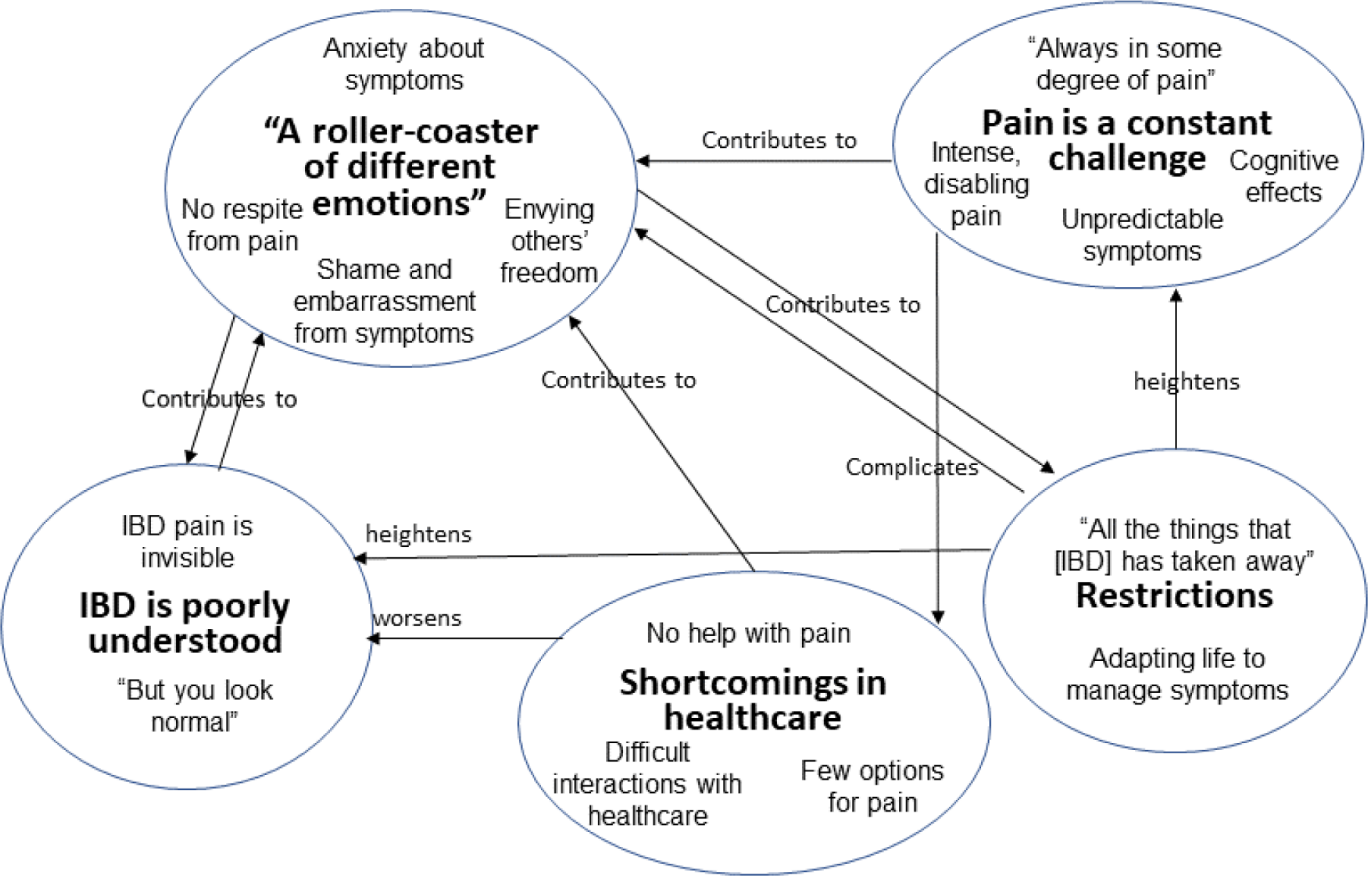
Thematic map with connections between themes. IBD, inflammatory bowel disease.

Table 2 provides an overview of the themes and subthemes with occurrence across participants. Figure 1 is a thematic map showing connections between themes.

**Table 2:** Overview of themes and subthemes, with occurrence across participants

Themes	Subthemes N endorsing	Examples
*A rollercoaster of emotions*	1.1. Anxiety about symptoms 30	*It was making me actually be sick, the worries about obstruction, it’s always really frightening when it happens*. (F11) *You have that fear and anxiety of going out somewhere without knowing… if there’s a toilet available, where the nearest disabled toilet is, or you know, there is the anxiety of going into a public toilet… and having to do your business in a place where everyone can hear*. (F15)
	1.2. No respite from pain 30	*So being in pain is extremely depressing, and I think I linked that to not being able to control the pain… and not understanding why*. (F14) *When it’s really bad, I'm sort of sitting on the sofa feeling a bit depressed… I don't feel like there’s any solution for it at the moment… I've sort of got to a stage of where I’m sort of not expecting anyone to find a solution…* (F1)
	1.3 Shame and embarrassment at symptoms 30	*There are a lot of people who are like, ^“^Ooh, that’s gross and then that’s embarrassing”, making you think “Ohh God, I'm gross”… It’s not my fault… there’s a lot of stigma about it*. (F13) *I'm 42 years old in a full-time job. I should not be sleeping three times in a day. I've almost gone back to like my baby state. I always want to wrap up. And then I need my various sleeps throughout the day and to top it all off, I'm … fed like baby food through a tube*. (M6)
	1.4. Envying others’ freedom 15	*I think hearing about what other people do can be very frustrating as well… I feel like it’s not because I wouldn't want to do stuff, it’s that I can't* (M13)
Pain a constant challenge	2.1. Always in some degree of pain 26	*There’s always that uncertain underlying baseline level discomfort and cramps and that seems to be like the main feature, even when it’s not really bad*. (M4) *Each time I'm having a flare-up… the pain… It’s just really long. It just makes your days feel like they go on forever*. (F4)
	2.2. Intense/disabling pain 30	*I get excruciating pain in the lower abdomen…*.[it] *takes your breath away. It can double me over… I can imagine it feels very similar to being stabbed. Like I can't breathe*. (M10)
	2.3. Cognitive effects 30	*Tingly is the way that I explain how the pain makes my brain feel. It’s like all tingly and numb*. (F4)
	2.4. Unpredictable symptoms 30	*Things that I plan, I tend to really look forward to doing them. So then not being able to do them because I'm in pain for whatever reason or because I'm too tired because of the, you know, related things, I find that very annoying… unpredictably getting in the way of life, I guess*. (F14)
Restrictions	3.1. All the things [IBD] has taken away 30	*Before I had Crohn’s, I had a busy social life… but now because of me, my social life and our social life has dropped off*. (M6) *It does sort of rip your life apart in the kind of social and psychological aspect, it rips apart your plans for life… rips apart relationships, I’ve lost quite a few friends and family members, those relationships ended because I was ill and they couldn't deal with it… hopes and dreams, it rips those up too*. (F9)
	3.2. Adapting life to manage symptoms 22	*I was in* [online] *meetings, and I’d turn my camera off and, in some meetings, I was going to the toilet twice in an hour-long meeting. So, you know, just hoping, praying that nobody had asked me a question*. (F10)
Shortcomings in healthcare	4.1. No help with pain 20	[Pain is] *often very overlooked compared with other symptoms… it’s always kind of shocking to me the amount of times that the response is like, ^“^Oh well, yeah, you would have that”… they don't then address the fact that you're in constant pain*. (F2)
	4.2. Difficult interactions with healthcare 12	*Over the last year when I was unwell… my flare was not showing up in my bloods, so they were very much like ^“^all this is probably just a bit of anxiety”, I mean they love to throw that label around and I know that just in general with women’s health, so that’s just their go-to you know, it’s kind of the equivalent of 50 years ago saying you got hysteria, and I'm sure that that probably makes me more anxious because I'm like, ohh, and now I'm sore and now they don't even believe me that I'm sore*. (F12) *I was told I had IBS: we had our final meeting with the gastro guy, his exact words were ^“^You’ll be one of the boys going out for beers with your mates, so everyone will have a few beers and a curry, you’ll just be one of the guys that after having a curry will suffer the next day. “Don’t eat curry.”* (M10)
	4.3. Few options for pain 30	*My consultant is reluctant to try me on another* [medication] *‘cause I've done four… you'll eventually run out if you keep going, and his words to me were ^“^Let’s not rock the boat”. I can kind of see his point, but I want the pain taken away*. (M6)
IBD is poorly understood	5.1. IBD pain is invisible 25	*You got initially diagnosed, and everyone’s sort of, you know, “Hey, how you doing?”, and then after a while, “Well, you’re on medication, you must be better”… and just because I don't talk about it, they don't really realise I'm still in pain, you know?* (F1).
	5.2. But you look normal 26	*Crohn’s disease is, what they say, an invisible illness… It has a huge stigma because… pain, it’s very difficult to prove*. (F7) *So, when I am socialising, or working, or just being in public, being with other people, I have to mask the pain and I have to pretend like I'm not feeling this 24/7. I don't want to be known as that girl that’s always in pain, or that girl with the Crohn’s*. (F1)

Direct quotations are shown in italics, with participant number following.

F, female; IBD, inflammatory bowel disease; M, male.

#### “A rollercoaster of different emotions” (M4)

The first theme speaks to the emotional toll expressed by all participants in some form.

##### Anxiety about symptoms

All participants described anxiety and fear about symptoms, particularly the meaning and implications of pain. Was it a medical emergency such as obstruction? Should they seek immediate healthcare? Anxieties were also expressed about the future progression of the disease, prolonged flares and prolonged recovery and about vulnerability to infection while immunosuppressed. A further worry concerned difficulties finding a toilet sufficiently urgently when in public places and ensuring privacy. All these added to the stresses of daily life and could therefore worsen pain and other symptoms.

##### No respite from pain

This subtheme was associated with expression of sadness and hopelessness by all participants in relation to pain unpredictability and uncontrollability, even with analgesics.

##### Shame and embarrassment at symptoms

All participants expressed embarrassment and shame in relation to IBD symptoms. Stigmatising judgements by others were internalised by some; others believed that they managed IBD poorly compared with others and that reflected poorly on them. Many had made compromises in their lives and life goals which meant that they fell short of their own or others’ expectations.

##### Envying others’ freedom

Unfairness and resentment about restrictions imposed by IBD symptoms was expressed by about half the participants, exacerbating feelings of being isolated from normal social activities. A few vigorously rejected the term disability: *Someone said, “You’re disabled, oh poor you”, and it’s not ‘poor me’. I've got a condition. I live with it. That’s who I am*. (M9)

### Pain a constant challenge

All participants described in some way the struggle with pain and other symptoms of IBD.

#### “Always in some degree of pain” (M1)

All but four participants had constant pain, regardless of disease remission, varying from a background manageable level to all-consuming peaks of pain with no relief available.

#### Intense/disabling pain

All participants provided descriptions of their pain, often using vivid and violent metaphors or similes involving damage or destruction, others full of uncertainty and possible threat.

#### Cognitive effects

All participants described other symptoms of IBD, particularly fatigue and ‘brain fog’, either or both impairing concentration on work, social interactions or completion of everyday tasks.

#### Unpredictable symptoms

The unpredictability of symptoms added to difficulties in planning and in realising plans, leaving participants feeling they were not in control. Intense pain could occur suddenly, interrupting any activity and efforts to identify triggers, particularly in food, which rarely proved reliable.

### Restrictions

Many restrictions resulted from attempts to control symptoms or planning to manage them when out of the house.

#### “All the things that (IBD) has taken away” (F11)

Participants expressed a strong sense of loss as a result of these restrictions: *So, if you think of the world, if you like, the world where everyone lives, when I have pain, mine shrinks* (F11). Social life in particular was affected by frequency and urgency of needing a toilet and, for some, by dietary constraints and many participants felt guilty about shared restrictions for partners and family members. A sense of loneliness and isolation was common, particularly when participants felt that those around them did not understand their needs, and because pain caused individuals to withdraw socially. Sleep was also disrupted by the need to use the toilet several times per night.

Long-term life goals had been abandoned as impossible to pursue, given the restrictions; many concerned work and career progression, although some had employers who had accommodated their needs. One participant had given up the hope of having children: *I can barely take care of myself, so how would I take care of some kid, you know?* (F9). Several participants described how IBD had adversely affected important relationships, including partners, with some feeling unable to form sexual relationships.

#### Adapting life to manage symptoms

The difficulty of identifying and avoiding triggers was described by 22 participants, mostly through dietary experiment, sometimes resulting in a bland and narrow diet and reduced pleasure in eating. Many found some control in timing of meals or ate ‘forbidden’ foods on days when they had unrestricted access to a bathroom. For some, working from home (during and since COVID-19) had made life easier.

Coping with pain also took trial and error and effort. Several participants had developed breathing exercises, physical movement or distractions; others sought comfort and support from social contact, religious practice or therapy, and a few self-medicated, including with cannabis.

#### Shortcomings in healthcare

The fourth theme described interactions with healthcare, mostly but not all unsatisfactory.

#### No help with pain

Inadequate understanding of pain was reported by 20 participants and many felt unsupported in managing pain with analgesics as the only suggestion despite potential adverse effects on the gut. Several participants had experienced delayed diagnosis or misdiagnosis; some had received conflicting advice from clinicians about medication, diet and symptom management. One was advised that CD would settle to a minimal level by his mid-20s and was deeply disappointed that this proved incorrect. *It took a long time for me to get into my head that actually this isn't going to happen*. (M13)

#### Difficult interactions with healthcare

12 participants described having to plead with clinicians for care, with symptoms dismissed as anxiety or ‘psychosomatic’, particularly when tests showed IBD to be in remission. This was associated for some with delayed diagnosis, and frustration as well as relief when the eventual diagnosis of IBD was made, since symptoms had progressed and treatment options narrowed during the delay. Several had felt invalidated when describing their pain and lost trust in their clinicians.

#### Few options for pain

Treatment for IBD was mentioned by all participants: finding effective treatment for changeable symptoms was difficult, and the balance of adverse effects against benefits was often hard to estimate. Given the few treatment options available for IBD, many participants were concerned about running out of options, with their clinicians reluctant to progress through medications until they proved ineffective or intolerable, but this left symptoms poorly controlled for long spells. Many preferred to be at home when they were in severe pain rather than attend a hospital for care, but at the same time were concerned about not acting sufficiently promptly in an emergency such as intestinal obstruction.

### IBD is poorly understood

The final theme draws together ways in which IBD pain made participants feel poorly understood and different from their peers and the challenges that arose from this.

#### IBD pain is invisible

25 participants felt that others did not understand their experience of living with IBD, particularly how pain affected them, sharing examples of unhelpful comments from others, often people close to them. Others spoke of stigma about bowel function, particularly in the workplace.

#### “But you look normal” (M11)

The invisibility of IBD and of pain necessitated disclosure, with the risk of stigma and social disapproval; 26 participants described difficult interactions. Some had felt expected to ‘prove’ the severity of their disease and of pain to family, friends or colleagues, and how much information to provide was a common dilemma. Many participants described masking IBD symptoms, particularly pain, from others, to meet social or work expectations; a few did not want their identity to be subsumed under the diagnosis.

### Addendum

Comments were received from researchers, clinicians and an expert by experience on these results: none resulted in any changes to thematic analysis, but they contributed to the discussion.

## Discussion

### Main findings

Pain in IBD dominated one theme and informed all four others, particularly *Rollercoaster of emotions*, and *Shortcomings in healthcare*. As described by Sweeney *et al*,^7^ participants contextualised their pain in relation to other symptoms, but a few by their perceptions that their gender or ethnicity led to inequitable healthcare. The sense of being socially excluded, actively by stigma and passively by poor understanding, emerged very strongly, and, when it extended to healthcare, this was a particular source of distress and hopelessness. Pain and other IBD symptoms can disrupt fundamental human needs such as belonging and autonomy^26^ and, for many, COVID-19 increased their isolation because of ‘shielding’ from infections.

These themes resonate with other qualitative studies, in terms of the powerful emotions associated with pain,^7 27^ the importance of the psychosocial impact of symptoms^28^,^31^ including internalised stigma,^32^ and the abandonment or amendment of life goals, at a cost to self-esteem.^33^ Social concerns were particularly prominent: how, when and to whom to disclose symptoms, with anticipated stigma and embarrassment or disclosure being forced by symptoms^34^ and the confusion this could create after successful concealment. Women in particular expressed sensitivity about the conflict of symptoms with gender expectations^35^ and also about others’ erroneous attributions of their restricted diet to slimming or eating disorder and frequent visits to the bathroom as pregnancy sickness.

While the consistency of these results may seem to offer little novel to our understanding of IBD pain, they contrast with the model of chronic pain that is often extended to visceral pain from its widespread use in musculoskeletal pain. We found little evidence of life plans, on a daily basis or a longer timescale, being dominated by concerns about exacerbating pain and fears of irreparable damage, resulting for the more cautious in a lifestyle disabled more by beliefs than by pain.^36^ Unpredictable onset and severity of symptoms—pain, fatigue, diarrhoea and urgency—imposed restrictions that left people deprived of normal social pleasures, but no participant believed that normal household, social and work activities would harm them. Anxiety can, of course, constitute a major or minor contributor to urgency, as can gut-brain mechanisms such as those active in irritable bowel syndrome: both can seriously impair quality of life.^37^ Fears about flares and about progression of IBD were met with attempts to control symptoms, often by food restrictions, or were addressed in healthcare settings. Participants did describe struggling to make meaning of the pain, in particular whether sudden severe pain was a medical emergency or manageable at home, and none expressed confidence in identifying and avoiding ‘triggers’. However, this is more consistent with a commonsense model of self-regulation in illness^38^ that addresses how individuals understand and make sense of their illness, including efforts to control symptoms, than the chronic pain model of catastrophic misinterpretation of pain and associated avoidance of activity.

### Limitations

We recognise that our focus on pain elicited material mainly about pain, but beyond the initial question, the interview process was far less directive than many. This is both an advantage, in allowing participants to decide what to discuss where the breadth of possible topics is not known, and a disadvantage, in that the same topics were not covered in all interviews as they would be with predetermined questions. We therefore cannot determine whether topics not addressed were of no importance or simply of lower priority than those selected by participants for elaboration. Conducting interviews online may have excluded potential participants less comfortable with using the technology, but it allowed us to include participants from anywhere in the UK, regardless of distance or travelling difficulty. Perhaps more important is that we recruited through an IBD charity which provides support and information, potentially mitigating fears and dispelling poorly informed anxieties. The population may have been better educated and more confident in managing IBD pain and other symptoms than a clinical population; this concern was raised particularly by a pain clinician commentator. We did not systematically collect any information about their medical history or current health, other than pain severity and interference. However, many participants were currently actively receiving healthcare, and their average pain and disability was at a moderate level (similar to that noted in a clinical population),^39^ so there is some overlap with clinical populations.

### Implications

As psychological support and interventions are introduced into gastrointestinal care, as indicated from research findings and desired by patients,^2729 40^,^42^ pragmatically coherent models are required to assess and formulate patients’ difficulties and needs. We suggest that, while it is undoubtedly important to identify anxieties that can be resolved by accurate information and behavioural experiment (eg, with diet) and to address avoidance of valued activities that is based on overestimates of threat, as in cognitive-behavioural treatment for other chronic pain, there should be no assumption that those are central for all. Models need to be better embodied,^43^ to understand brain-gut axis elements in symptom appraisal and to draw on areas of psychological intervention for stigma and disclosure. Further research could directly test the extent of catastrophising and the strength of connection between pain-related fears and activity avoidance, using standard questionnaires, to compare with clinical musculoskeletal chronic pain populations.

## Conclusions

There is a clear need for pain to be better acknowledged and addressed in medical care of IBD^1 42^ and in research studies, since it has wide-ranging impact on the lives of people with IBD. Medical care could helpfully include establishing algorithms for self-management of non-emergency increases in pain and building patients’ confidence in identifying signs that require medical attention; support for systematic experiments in identifying triggers to pain increase and flares.^41 42 44^ Clinicians could better recognise that validation of pain and of its emotional toll strengthens trust in care,^44 45^ thereby improving outcomes. Onward referral from medical to psychological services for help with self-management is most acceptable when medical and psychological services are connected. We consider that our findings cast sufficient doubt on the fear and avoidance model applied to IBD pain that, given the disappointing results of current psychological interventions for pain applied to IBD,^40^ revision of content to address concerns described here would be an appropriate next step.

## Supplementary material

10.1136/bmjgast-2025-001866 online supplemental file 1

10.1136/bmjgast-2025-001866 online supplemental file 2

## Data Availability

Data are available upon reasonable request.
