# Supplementary material for: Pain experience of people with inflammatory bowel disease: a qualitative study
Source: BMJ Open Gastroenterol. 2025 Sep 5;12(1):e001866. doi: 10.1136/bmjgast-2025-001866 (PMC12414221; doi:10.1136/bmjgast-2025-001866)

**Researcher positionality statements**

Three researchers (CB, AA, HM) conducted the interviews (18, 3 and 9 interviews respectively), and a fourth (AW) was involved in data analysis. Throughout the project, researchers discussed interview findings and process, aiming for reflexive processing of data, considering how their beliefs and concerns might influence their reading and analytic decisions. It is essential for researchers to reflect on their positionality when conducting qualitative research (Barker & Pistrang, 2005).

CB is a white, British, middle-class female in her late 20s, completing the research as part of her Clinical Psychology Doctorate. Professionally, the researcher had not worked in IBD, but had experience in other areas of psychology applied to medicine. She has a diagnosis of Crohn’s disease, but did not disclose this to participants unless directly asked why she decided to conduct this study, which occurred only in one interview with a male participant. During the research, she kept a reflective journal and received regular supervision from AW. She approached the interviews with a belief that visceral pain differs from musculoskeletal pain, can be debilitating, and has been neglected in the research literature and, therefore aimed to amplify participants’ experiences.

AA is a research assistant, a British-Bangladeshi female in her early 20s with a working-class upbringing, working with AW on the ADVANTAGE study to understand pain experience in various visceral diseases, including IBD. She was trained in GEM during her undergraduate degree and used her training to conduct interviews for this project. She has experience with chronic pain but chose not to disclose this to interviewees; if asked by interviewees at the end of the interview, she briefly alluded to her experiences. HM is a research assistant with experience in qualitative research and a MA in Art History and Psychology. This was her first project researching IBD, and she considers the biopsychosocial model to be the most convincing pain model to date. AW is an academic and clinical psychologist, with over 35 years’ experience working in chronic pain. While she has used the fear and avoidance model in academic and clinical work, she considers it to capture only part of the chronic pain experience, even in musculoskeletal pain; she embarked on this research with open questions about whether the model described the visceral pain experience well. She does not have chronic pain.

**Examples of GEM Grids**


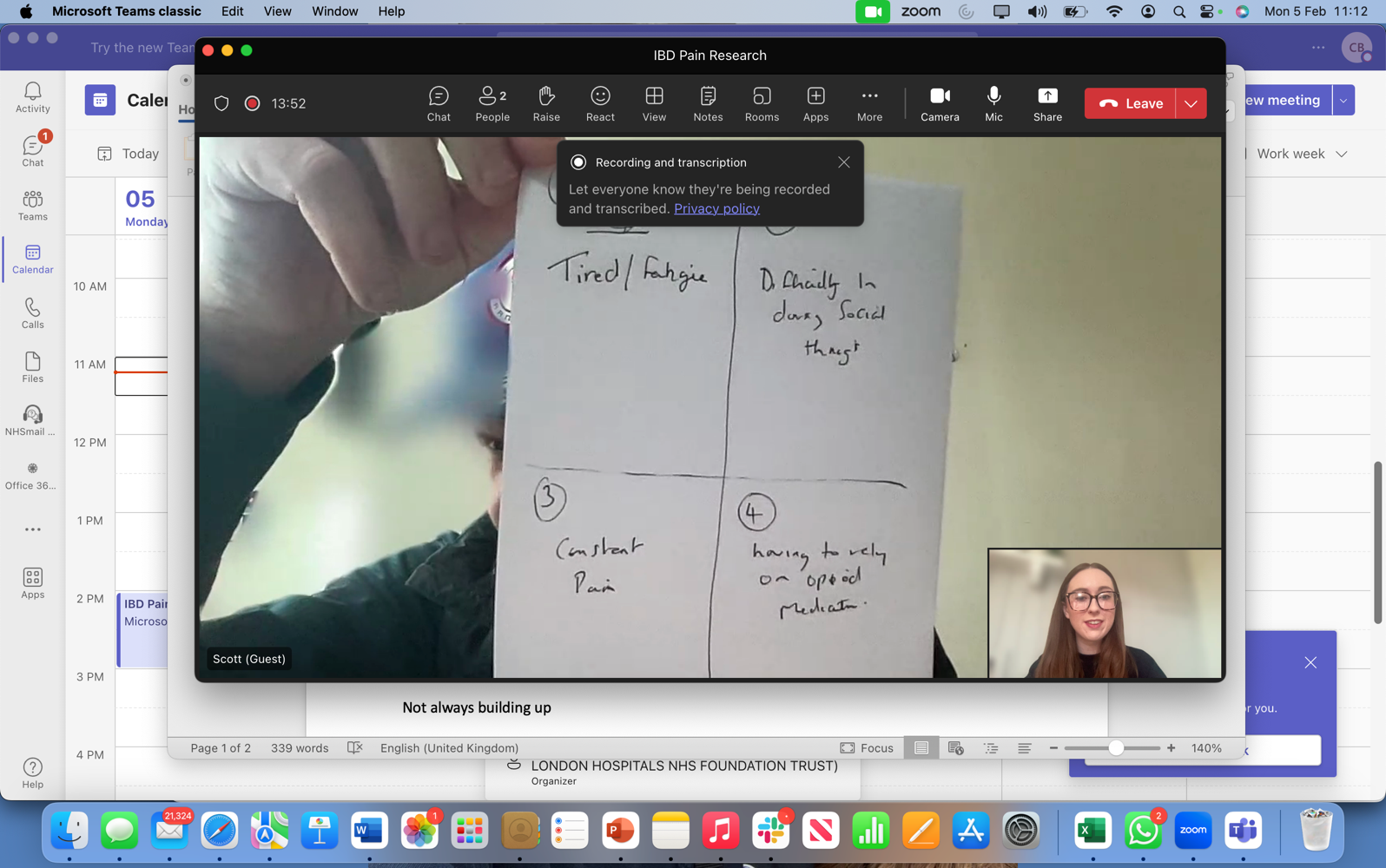


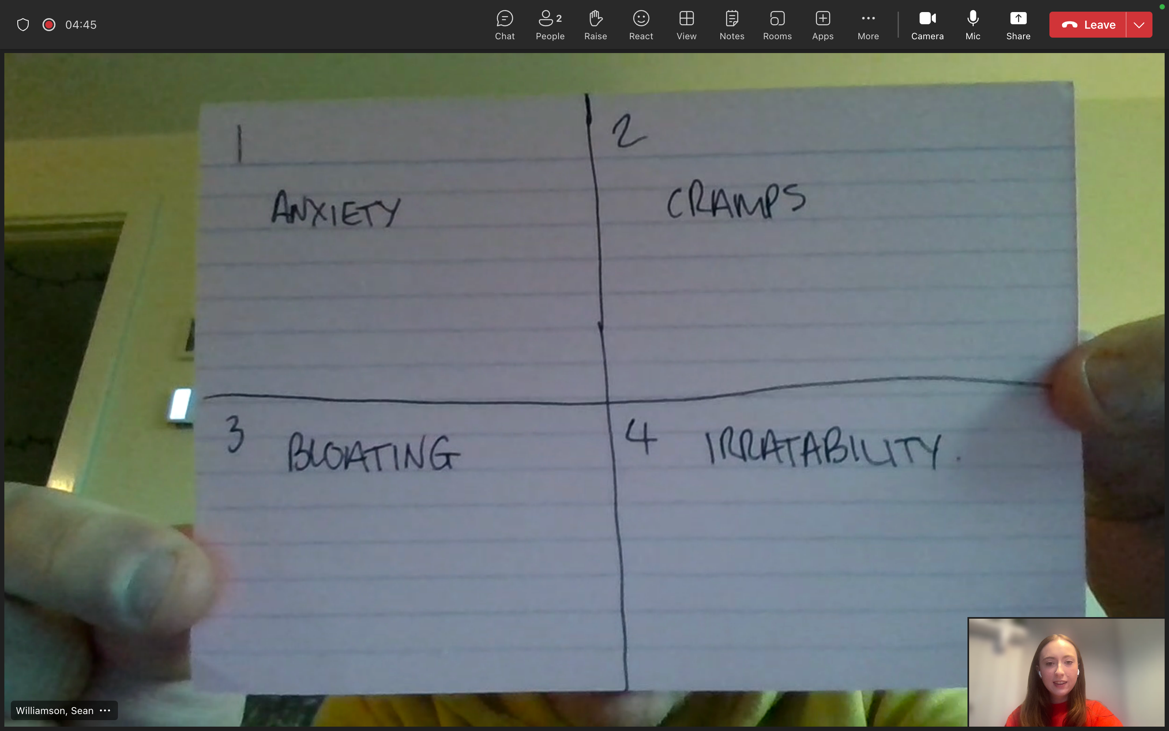

Supplement: online supplemental file 1 [file bmjgast-12-1-s001.docx]
