# Supplementary material for: Pain experience of people with inflammatory bowel disease: a qualitative study
Source: BMJ Open Gastroenterol. 2025 Sep 5;12(1):e001866. doi: 10.1136/bmjgast-2025-001866 (PMC12414221; doi:10.1136/bmjgast-2025-001866)
Supplement: online supplemental file 2 [file bmjgast-12-1-s002.docx]

**COREQ checklist**

| ***Item*** | ***Guide questions/description*** | ***Reported on page #*** |
| --- | --- | --- |
| ***Domain 1 Research team & reflexivity*** | | |
| *Personal characteristics* | | |
| 1. Interviewer/ facilitator | Which author/s conducted the interview or focus group? | CB, AA, HM: stated in Methods p6 |
| 2. Credentials | What were the researcher’s credentials? E.g. PhD, MD | In Methods p7 & supplementary data |
| 3. Occupation | What was their occupation at the time of the study? | In Methods p7 and supplementary data |
| 4. Gender | Was the researcher male or female? | All four female: Methods p7 and supplementary data |
| 5. Experience & training | What experience or training did the researcher have? | In Methods p7 |
| *Relationship with participants* | | |
| 6. Relationship established | Was a relationship established prior to study commencement? | No relationship beyond recruitment correspondence: p7 & supplementary data |
| 7. Participant knowledge of the interviewer | What did the participants know about the researcher? e.g. personal goals, reasons for doing the research | Participants had no information about researcher other than aims of research in participant information: p6-7 |
| 8. Interviewer characteristics | What characteristics were reported about the inter viewer/facilitator? e.g. Bias, assumptions, reasons and interests in the research topic | Interviewer characteristics described in Methods p7 and in supplementary data |
| ***Domain 2: study design*** | | |
| *Theoretical framework* | | |
| 9. Methodological orientation and Theory | What methodological orientation was stated to underpin the study? e.g. grounded theory, discourse analysis, ethnography, phenomenology, content analysis | Design & setting: p5 |
| *Participant selection* | | |
| 10. Sampling | How were participants selected? e.g. purposive, convenience, consecutive, snowball | Volunteers from advertisement on website of Crohn’s & Colitis UK charity: see Design and setting p5-6 |
| 11. Method of approach | How were participants approached? e.g. face-to-face, telephone, mail, email | Email correspondence: see Procedure p6 |
| 12. Sample size | How many participants were in the study? | 30: see Participants p5 |
| 13. Non-participation | How many people refused to participate or dropped out? Reasons? | No refusals or drop-outs: see Results p8 |
| *Setting* | | |
| 14. Setting of data collection | Where was the data collected? e.g. home, clinic, workplace | Online - participants at home: see Procedure p6 |
| 15. Presence of non-participants | Was anyone else present besides the participants and researchers? | No: p6 |
| 16. Description of sample | What are the important characteristics of the sample? e.g. demographic data, date | 15 women, 15 men: mixed ages, ethnicities, years since diagnosis. See Table 1. |
| *Data collection* | | |
| 17. Interview guide | Were questions, prompts, guides provided by the authors? Was it pilot tested? | Guide created and trained by originators of GEM; no further prompts / questions added. Pilot on first participant generated no changes, so included in sample. P5-6 |
| 18. Repeat interviews | Were repeat inter views carried out? If yes, how many? | No repeat interviews. |
| 19. Audio/visual recording | Did the research use audio or visual recording to collect the data? | Video recording of interviews kept for correction of transcription and then deleted upon completion: see Interviews p7. |
| 20. Field notes | Were ﬁeld notes made during and/or after the interview or focus group? | Brief notes taken before and after interviews: see Interviews p7. |
| 21. Duration | What was the duration of the inter views or focus group? | Scheduled for one hour, see Procedure p6 and most approximated one hour, see Results p8. |
| 22. Data saturation | Was data saturation discussed? | Sample size advised by originator of GEM; salience of themes and subthemes shown in Table 2. |
| 23. Transcripts returned | Were transcripts returned to participants for comment and/or correction? | No. |
| **Domain 3: analysis and ﬁndings** | | |
| *Data analysis* | | |
| 24. Number of data coders | How many data coders coded the data? | Two: CB and AW: see Data Analysis p7-8. |
| 25. Description of the coding tree | Did authors provide a description of the coding tree? | Coding frame available from authors on request. |
| 26. Derivation of themes | Were themes identiﬁed in advance or derived from the data? | Derived from data: see Data Analysis p7-8. |
| 27. Software | What software, if applicable, was used to manage the data? | NVivo: see Data Analysis p7. |
| 28. Participant checking | Did participants provide feedback on the ﬁndings? | Participants were not asked for feedback, but we invited comments from experts in the field: p8 & Acknowledgements. |
| *Reporting* | | |
| 29. Quotations presented | Were participant quotations presented to illustrate the themes/ﬁndings? Was each quotation identiﬁed? e.g. participant number | Yes: see Table 2 |
| 30. Data and ﬁndings consistent | Was there consistency between the data presented and the ﬁndings? | In Results pp8-15, and Discussion pp15-18. |
| 31. Clarity of major themes | Were major themes clearly presented in the ﬁndings? | Yes, in Results pp8-15, in Table 2 and in thematic map Fig 1. |
| 32. Clarity of minor themes | Is there a description of diverse cases or discussion of minor themes? | Yes: Table 2 shows salience of subthemes. |
